# Clinical features and genetics in non-5q spinal muscular atrophy caused by acid ceramidase deficiency

**DOI:** 10.25122/jml-2021-0147

**Published:** 2021

**Authors:** Mihaela Axente, Elena-Silvia Shelby, Andrada Mirea, Corina Sporea, Mihaela Badina, Liliana Padure, Daniela Adriana Ion

**Affiliations:** 1. Department of Pathophysiology, Carol Davila University of Medicine and Pharmacy, Bucharest, Romania; 2. Dr. Nicolae Robanescu National Neurorehabilitation Center for Children, Bucharest, Romania; 3. Department of Balneophysiokinetotherapy, Carol Davila University of Medicine and Pharmacy, Bucharest, Romania

**Keywords:** spinal muscular atrophy, Farber disease, ASAH1 gene

## Abstract

Spinal muscular atrophy (SMA) is a spectrum of genetically and clinically heterogeneous diseases leading to the progressive degeneration of peripheric motor neurons with subsequent muscle weakness and atrophy. More than 95% of the cases of SMA are represented by homozygous mutations of the SMN1 gene (5q-SMA). Because this disease represents the leading cause of death due to a genetic cause and due to the availability of genetic therapies which can now save the life of the patient and stop the progress of the disease, early diagnosis is crucial. This report presents the case of a 13-year-old patient admitted to our hospital in 2018 who presented a phenotype typical to 5q-SMA. Next-generation sequencing (NGS) and Sanger sequencing of the SMN1 gene were performed, and a negative result was obtained. Consequently, we continued testing using whole-exome sequencing and discovered three mutations in the ASAH1 gene (one pathogenic and two variants of uncertain significance). Pathogenic mutations in the ASAH1 gene are responsible for spinal muscular atrophy with progressive myoclonic epilepsy (SMA-PME) and Farber disease, which overlapped with our patient’s phenotype. Currently, there are 45 SMA cases caused by mutations in the ASAH1 gene reported worldwide; however, the present case is the first reported in Romania.

## INTRODUCTION

Spinal muscular atrophy (SMA) is a group of inherited genetically and clinically heterogeneous syndromes with various inheritance patterns: autosomal dominant, autosomal recessive, or X-linked [1, 2]. The common feature of this progressively degenerative spectrum of genetic diseases is represented by the degeneration of the motor neurons in the anterior horns of the spinal cord and brainstem, leading to the destruction of the alpha motor cells and, consequently, to the atrophy of various muscular groups in the body [1–3]. The manifestations are very heterogeneous: the onset can be anytime between childhood and adulthood, and various degrees of severity can be present (patients can be mildly symptomatic or can die in infancy), and each syndrome can affect certain muscle types (proximal or distal) [2, 3].

About 95% of the cases of SMA are caused by homozygous deletions in exons 7 and 8 of the SMN1 gene (5q13) [4]. Consequently, this disease was called 5q-SMA [1, 5, 6]. The rest of approximately 5% of the cases are caused by mutations in other various genes, this spectrum being called non-5qSMA [1]. Because oftentimes, the clinical manifestations overlap, the only certain diagnosis mode is genetic testing [2].

Globally, 5q-SMA is the leading cause of death due to a genetic disorder and, after cystic fibrosis, the second most common autosomal recessive disorder [7]. Moreover, currently, 5q-SMA has become one of the first treatable neuromuscular genetic disorders, with therapies such as insertion of the wild-type gene using a viral vector or splice correction via administration of modified antisense oligonucleotides being able to save the life of the patient and stop the formation of the known debilitating sequelae specific to this disease upon early administration [5]. Therefore, early and precise diagnosis of 5q-SMA is essential.

Currently, 16 genes and one unresolved locus have been found to be associated with non-5qSMA [8]. Among them, ASAH1, situated in the 8p22 locus, encodes the ASAH protein (N-acylsphingosine amidohydrolase 1, N-acylsphingosine deacylase or acid ceramidase), an enzyme involved in the cleavage of ceramide into sphingosine and a free fatty acid at the lysosomal level, as well as in the synthesis of ceramide from the above-mentioned components under different pH conditions [8–14].

Biallelic pathogenic mutations in ASAH1 are involved in the production of two diseases with an autosomal recessive inheritance mode whose phenotype can sometimes overlap: Farber lipogranulomatosis, also known as Farber disease (FD), and spinal muscular atrophy with progressive myoclonic epilepsy (SMA-PME) [9–12, 14, 15]. Up to now, there are less than 200 cases of FD and SMA-PME reported in the literature [16].

Other phenotypes caused by homozygous mutations in the ASAH1 gene are spinal muscular atrophy without epilepsy and progressive adult-onset brachydactyly due to osteolysis [15].

Farber disease is a very rare lysosomal disease [16]. Classic Farber disease has a life expectancy of less than two years [15]. The disease starts in the first weeks of life with pain, swelling, and progressive deformity (contractures) of the major joints as well as painful, palpable subcutaneous nodules at the pressure points and joints [15, 16]. Most patients have a hoarse voice caused by granulomas of the larynx and epiglottis [15].

Nevertheless, the disease has variable expressivity, and the affected patients can present very mild symptoms in rare cases, which often lead to misdiagnosis or a very late diagnosis [16].

SMA-PME was described for the first by Jankovic and Rivera in 1979 as a clinical syndrome associating lower motor signs and progressive myoclonic epilepsy. SMA-PME starts between the age of three and seven and is caused by the degeneration of the lower spinal motor neurons. The disease starts with proximal weakness and atrophy of the lower limbs with onset around the age of five years, followed by progressive intractable myoclonic seizures in late childhood with jerking of the upper limbs, myoclonic status, action myoclonus and eyelid myoclonus [10, 15]. Generalized tremor, cognitive decline, and sensorineural hearing loss are also among the manifestations of this disease [10, 15]. The prognosis is very poor, with death in adolescence [10, 15].

Although FD and SMA-PMA are generally considered two phenotypically distinct diseases, Teoh *et al*. have described in 2016 the case of a nine-year-old girl who presented a novel clinical phenotype, with polyarticular arthritis followed by SMA symptoms [17]. Upon genetic testing, the patient was found to have compound heterozygous mutations in the ASAH1 gene [17]. Thus, the authors demonstrated that FD and SMA with progressive myoclonic epilepsy are not always distinct diseases but belong to an evolving phenotypic spectrum [17].

### Pathogenesis

Ceramides and their metabolites participate in various cellular mechanisms as lipidic mediators. Ceramides are cleaved in the lysosomes by acid ceramidase into sphingolipids and fatty acids. In FD, the level of acid ceramidase is much lower compared to SMA-PME. The different phenotypes caused by mutations in the ASAH1 gene can be explained by the mutational effect on the final protein product [18].

## CASE REPORT

We present the case of a 13-year-old boy admitted to our hospital in September 2018 for severe respiratory distress, a global motor deficit with the lower limbs more affected than the upper limbs, motor regression, axial hypotonia with poor control of the head, muscle strength of 2–3/5 on the Medical Research Council’s scale (MRC) for the upper limbs and 2/5 on the MRC scale for the lower limbs, generalized severe muscular atrophy, retractions of the elbow and knees, more obvious in the distal segments.

The patient comes from a non-consanguineous twin pregnancy with term birth and no hypoxic events. He has a 19-year-old healthy brother.

In the first year of life, the patient was hypotonic and had a moderate motor delay, sat after 1 year, and walked without support after 2 years. He never achieved running, climbing stairs, or jumping on one foot. His lower limbs have always been more affected than his upper limbs. He had his first neurological examination at the age of 4, where the lack of deep tendon reflexes, tongue fasciculations, neurogenic changes on electromyography, motor deficit with the lower limbs being more affected than the upper limbs and muscular atrophy were observed. Biopsy revealed neurogenic changes raising the suspicion of a motor neuron disease. Genetic testing for 5q SMA – multiplex ligation-dependent probe amplification (MLPA) and Sanger sequencing – was negative. Between 5 and 10 years of age, the evolution was slowly progressive. The proximal weakness became severe, and the patient developed severe generalized muscle atrophy, swallowing dysfunction and ventilatory restriction, severe contractures at all levels, progressive scoliosis, and a lack of deep tendon reflexes. Gastrostomy was performed in 2019. After the age of 10, he became a wheelchair user, and his motor regression was dramatic. In March 2021, spine surgery was performed, after which muscular atrophy became extreme and the weakness was generalized. The patient lost head control and only presented a few active movements in the upper limbs. He presented paradoxical breathing without requiring noninvasive ventilation or oxygen. He had no deep tendon reflexes and presented a dysphonia with a hoarse voice. The patient also had retraction of the mandibular joint with very limited movements leading to the incomplete opening of the mouth, subcutaneous nodules around the interphalangeal joints, with the upper limbs more affected than the lower limbs. He had no paroxysmal events such as myoclonus or other types of seizures.

Electroencephalography (EEG) showed frequent generalized slow waves with a duration of 1–2 seconds, rarely focalized, with the left side more affected than the right one, without any clinical manifestations. Electroneurography (ENG) showed mildly reduced amplitude of Compound Motor Action Potential (CMAP) but with an increased area (due to the reinnervation process). Sensory Nerve Action Potential (SNAP) had normal amplitude and velocities. Electromyography (EMG) showed chronic denervation changes with very large, polyphasic motor unit potentials (MUP) and incomplete recruitment.

No liver enlargement was seen following the abdominal ultrasound. Spinal X-ray showed severe thoracal scoliosis (Cobb angle >100 degrees) with thoracic deformity and restrictive respiratory dysfunction.

In order to elucidate the diagnosis, a dried blood spot (DBS) card was sent for sequencing and copy number variation (CNV) assessment (using MLPA) of the SMN1 gene. No clinically relevant variant was identified following SMN1 sequencing and MLPA.

In the presence of a negative SMN1 test, we continued the testing using the whole-exome sequencing method. Genetic testing identified three heterozygous mutations in the ASAH1 gene. The first identified mutation (NM_004315.5:c.458_459del) was a two-base pair deletion in position 458 of the coding region in exon 6 generating the substitution of tyrosine in position 153 of the protein to a stop codon – p.(Tyr153Ter), or p.(Tyr153*), leading to a premature stop codon and thus, to a truncated protein. This variant is reported as pathogenic (class 1) according to the American College of Medical Genetics and Genomics (ACMG) guidelines.

The second identified mutation (NM_004315.5:c.1226T>C) was the T to C substitution of nucleotide 1226 of the coding region in exon 14, generating the substitution of isoleucine in position 409 of the protein to threonine – p.(Ile409Thr). This variant has been reported as a variant of unknown significance according to ACMG.

The third identified mutation (NM_004315.5:c.35G>C) was the G to C substitution of nucleotide 35 of the coding region in exon 1, generating the substitution of arginine in position 12 of the protein to a stop codon – p.(Arg12Pro). This variant is reported as a variant of uncertain significance according to ACMG.

The patient received supportive treatment in our Center, including respiratory assessment. Currently, he only needs cough assist 3–4 times/day, does not need oxygen or noninvasive ventilation. He receives daily physical therapy, respiratory and general, daily occupational therapy, neurocognitive evaluation, and psychotherapy. He has no need for anticonvulsant drugs. A severe evolution was noted, and the patient’s prognostic is poor due to rapid motor regression with extremely severe, generalized muscular atrophy and retractions, almost without any active movements, together with respiratory involvement (chronic respiratory failure) and swallowing difficulties (bulbar involvement).

## DISCUSSION

Mutations in the ASAH1 gene, which has about 30 kb and contains 14 exons, are responsible for two autosomal recessive disorders: FD and spinal muscular atrophy with myoclonic epilepsy [9, 16, 19]. If the two diseases were thought to be distinct at first, recent data showed that they are part of a phenotypic spectrum with often overlapping symptoms [16]. There is no genotype-phenotype correlation, and surprisingly, two cases where a patient with FD and a patient with SMA-PME had the same mutation have been reported [20, 21].

According to Yu *et al*., in 2018, there were 43 cases of SMA-PME and 153 cases of FD reported since 1952. For SMA-PME, the mean age of onset was 5.8 years, and the mean age of death was 14.4 years [16].

Currently, there are 45 submissions of pathogenic, likely pathogenic, benign, variants of uncertain significance (VOUS) or presenting conflicting interpretation variants in the ClinVar database, among which 10 have been associated with SMA-PME and 35 with Farber Disease [22].

The mutation NM_004315.5:c.458_459del, reported as pathogenic according to the recommendations of ACMG [23], consists of a 2 base pair deletion in position 458 of the coding region in exon 6, generating the substitution of tyrosine in position 153 of the protein to a stop codon – p.(Tyr153Ter), or p.(Tyr153*), leading to a premature stop codon and thus, to a truncated protein. The mutation is not present in the ClinVar database [22].

The mutation NM_004315.5:c.1226T>C generates the substitution of isoleucine in position 409 of the protein to threonine – p.(Ile-409Thr). This variant has been reported as a variant of unknown significance according to ACMG and is not reported in the ClinVar database.

The mutation NM_004315.5:c.35G>C generates the substitution of arginine in position 12 of the protein to a stop codon – p.(Arg12Pro) and has been classified as VOUS according to ACMG [24]. The mutation has been cited as associated with Rolandic epilepsy by Bobbili *et al*. in 2018 [25]. Due to the fact that its conservation is weak, it is possible that this variant is not damaging.

In the presented case, the onset was early, and the motor milestones have always been delayed (the patient never achieved certain milestones like running or climbing stairs). Subsequently, his motor regression was dramatic, with severe muscle weakness and extreme muscular atrophy, and retractions at all levels (the patient used a wheelchair from the age of 10). He had no clinical seizures but presented EEG epileptiform changes. His voice is hoarse, and he has subcutaneous nodules at the interphalangeal level; the upper limbs are more affected than the lower limbs. He has no painful or swollen joints. Having two out of three clinical criteria for FD, we consider this case quite particular, as patients with clinical aspects from both diseases are extremely rare. Nevertheless, the genetic results indicate that the mutation in exon 6 of the ASAH1 gene has been correlated with FD.

Unfortunately, due to this mutation and the different pathogenesis of this type of SMA, this case has no indication for gene therapy or antisense oligonucleotide therapy like classic SMA.

## CONCLUSION

SMA is a heterogeneous disease. The etiology of 5q and non-5q SMA can be challenging to distinguish as they have similar phenotypes. Cases of non-5q SMA caused by ASAH1 mutations are sporadic, with only 45 patients reported worldwide so far. We presented the first case ever reported in Romania of overlapping symptoms from SMA-PME and Farber disease caused by ASAH1 mutations. We believe our presentation is useful in furthering the knowledge about the SMA-PME/Farber disease spectrum.
